# TWELVE-YEAR CLINICAL TRAJECTORIES OF MULTIMORBIDITY IN OLDER ADULTS: A POPULATION-BASED STUDY

**DOI:** 10.1093/geroni/igz038.2271

**Published:** 2019-11-08

**Authors:** Davide Liborio Vetrano, Albert Roso-Llorach, Sergio Fernández, Concepción Violán, Graziano Onder, Laura Fratiglioni, Amaia Calderon-Larranaga, Alessandra Marengoni

**Affiliations:** 1 Aging Research Center - Karolinska Institutet, Stockholm, Sweden, Sweden; 2 Fundació Institut Universitari per a la recerca a l’Atenció Primària de Salut Jordi Gol i Gurina (IDIAPJGol), Barcelona, Spain, Spain; 3 Centro di Medicina dell’Invecchiamento, IRCCS Fondazione Policlinico “A. Gemelli” and Catholic University of Rome, Rome, Italy; 4 Aging Research Center, Department of Neurobiology, Care Sciences and Society, Karolinska Institutet and Stockholm University, Stockholm, Sweden; 5 Department of Clinical and Experimental Sciences, University of Brescia, Brescia, Italy

## Abstract

The scarce knowledge of multimorbidity development hampers the effectiveness of clinical interventions. We aimed to identify multimorbidity clusters, trace their evolution in a cohort of older adults, and detect the clinical trajectories of single individuals as they move among clusters over 12 years. Population-based study including 2931 persons 60+ with ≥2 diseases participating in the SNAC-K study. A fuzzy c-means cluster algorithm was used to group participants by disease patterns at baseline and follow-ups. Migration from one cluster to another was tracked over time, and the association between the clusters and mortality was tested. At baseline 52% of participants were classified into five clinically meaningful disease clusters: psychiatric and respiratory (5%), heart (9%), respiratory and musculoskeletal (16%), cognitive and sensory impairment (10%), and eye diseases and cancer (11%). The remaining 48% of participants (unspecified group) were grouped in any cluster at baseline but greatly contributed to the other clusters at follow-ups. Multimorbidity clusters that included cardiovascular and neuropsychiatric diseases presented a significantly higher mortality risk (odds ratios ranging 1.58–6.00) than the group not part of any clusters. Clusters characterized by cardiovascular and neuropsychiatric diseases included 25% of the study population at baseline and 28% of participants at six years, and they accounted for 51% of deaths at six years and 57% of deaths at twelve years. Multimorbidity clusters and clinical trajectories of older adults with multimorbidity show great dynamism over time. The multimorbidity clusters and trajectories identified in this study may help identifying groups with similar needs and prognosis.

